# Methodology for Low-Field, Portable Magnetic Resonance Neuroimaging at the Bedside

**DOI:** 10.3389/fneur.2021.760321

**Published:** 2021-12-10

**Authors:** Anjali M. Prabhat, Anna L. Crawford, Mercy H. Mazurek, Matthew M. Yuen, Isha R. Chavva, Adrienne Ward, William V. Hofmann, Nona Timario, Stephanie R. Qualls, Juliana Helland, Charles Wira, Gordon Sze, Matthew S. Rosen, William Taylor Kimberly, Kevin N. Sheth

**Affiliations:** ^1^Department of Neurology, Yale School of Medicine, New Haven, CT, United States; ^2^Neuroscience Intensive Care Unit, Yale New Haven Hospital, New Haven, CT, United States; ^3^Neuroscience Intensive Care Unit, Massachusetts General Hospital, Boston, MA, United States; ^4^Department of Emergency Medicine, Yale School of Medicine, New Haven, CT, United States; ^5^Department of Neuroradiology, Yale School of Medicine, New Haven, CT, United States; ^6^Athinoula A. Martinos Center for Biomedical Imaging, Massachusetts General Hospital, Charlestown, MA, United States; ^7^Department of Radiology, Harvard Medical School, Boston, MA, United States; ^8^Department of Physics, Harvard University, Cambridge, MA, United States; ^9^Department of Neurology, Massachusetts General Hospital, Boston, MA, United States

**Keywords:** neuroimaging, magnetic resonance imaging, neurology, bedside, low magnetic field

## Abstract

Neuroimaging is a critical component of triage and treatment for patients who present with neuropathology. Magnetic resonance imaging and non-contrast computed tomography are the gold standard for diagnosis and prognostication of patients with acute brain injuries. However, these modalities require intra-hospital transport to strict, access-controlled environments, which puts critically ill patients at risk for complications and secondary injuries. A novel, portable MRI (pMRI) device that can be deployed at the patient's bedside provides a needed solution. In a dual-center investigation, Yale New Haven Hospital has obtained regular neuroimaging on patients using the pMRI as part of routine clinical care in the Emergency Department and Intensive Care Unit (ICU) since August of 2020. Massachusetts General Hospital has begun using pMRI in the Neuroscience Intensive Care Unit since January 2021. This technology has expanded the population of patients who can receive MRI imaging by increasing accessibility and timeliness for scan completion by eliminating the need for transport and increasing the potential for serial monitoring. Here we describe our methods for screening, coordinating, and executing pMRI exams and provide further detail on how to scan specific patient populations.

## Introduction

Timely and accessible neuroimaging is crucial for accurate detection and treatment of neuropathology. High field magnetic resonance imaging (MRI) and computed tomography (CT) are the standard options for neuroimaging in most hospitals. Unless deemed life-threatening or hyper-acute, use of the MRI is subject to scanner availability as it is a shared resource and is balanced against the schedule. Additionally, these require patients to be temporarily disconnected from vital hospital equipment and transported to designated imaging suites, processes which are prone to significant time-delays. Such intra-hospital transport therefore has potential to jeopardize patient safety not only because it interrupts continuous monitoring, but also because it can lead to dangerous secondary injuries including airway displacement, oxygen desaturation, hypotension, hypertension, increased intracranial pressure, and hypoxia (1–6). Even with portable monitors, the risk of complications when transporting brain-injured patients is high since interventions cannot be performed in transit. Picetti et al. found 36% of brain injured patients experienced complications during transport (7). The MRI itself also poses risks of thermal burns or injury if a ferrous object enters the magnet and becomes a projectile (8), so critical care patients need to be screened very carefully before entering the magnet environment owing to the significant level of support equipment in this population. Physiologic monitoring during a conventional MRI is difficult as the sequences run on the MRI often interfere with monitor signal and the electrocardiogram cannot be reliably interpreted. Traveling to conventional MRI puts strain on the bedside nurse as they are absent from the unit for prolonged periods of time while accompanying these patients to imaging and may be caring for multiple patients. For these reasons, conventional MRI and CT are often inconvenient and inaccessible in the setting of critical illness.

Concerns with timely access to and the impact of intra-hospital transport, related to both safety and time, suggest an urgent need for point-of-care imaging. We have previously reported the use of a portable, low-field MRI device to obtain neuroimaging at the bedside. Our prior work has demonstrated the ability of this device to produce neuroimages that can detect primary injury like ischemic stroke and intracerebral hemorrhage, as well as other neurological sequelae and relevant biomarkers (9, 10). The pMRI device originally received clearance from the U.S. Food and Drug Administration in August 2020 and was incorporated into routine clinical care in the Yale New Haven Hospital (YNHH) Neuroscience ICU, Surgical ICU, and Emergency Department (ED). Massachusetts General Hospital (MGH) has been using the pMRI since 2021 in the Neuroscience ICU. The pMRI is unique from conventional MRI and CT, as well as portable CT (pCT) because it does not require specialized radiologic technicians to operate and avoids the use of harmful ionizing radiation. pMRI is considerably lower strength (64 mT) as compared to conventional MRI (1.5–3T), which minimizes the risk of thermal burns and ferrous projectiles. Furthermore, pMRI offers different pulse sequences, including T2-weighted imaging (T2W), fluid-attenuated inversion recovery (FLAIR), diffusion-weighted imaging (DWI), and T1-weighted imaging (T1W), which provide helpful tissue contrast for characterizing intracranial pathology. While initial deployment of the pMRI in the ICU has been described (9–12), this paper aims to expound the process of screening, coordinating, and executing pMRI brain scans to inform future users about the specific methodology and techniques that are required for integration into clinical settings.

## Materials and Equipment

**Figure 1:**
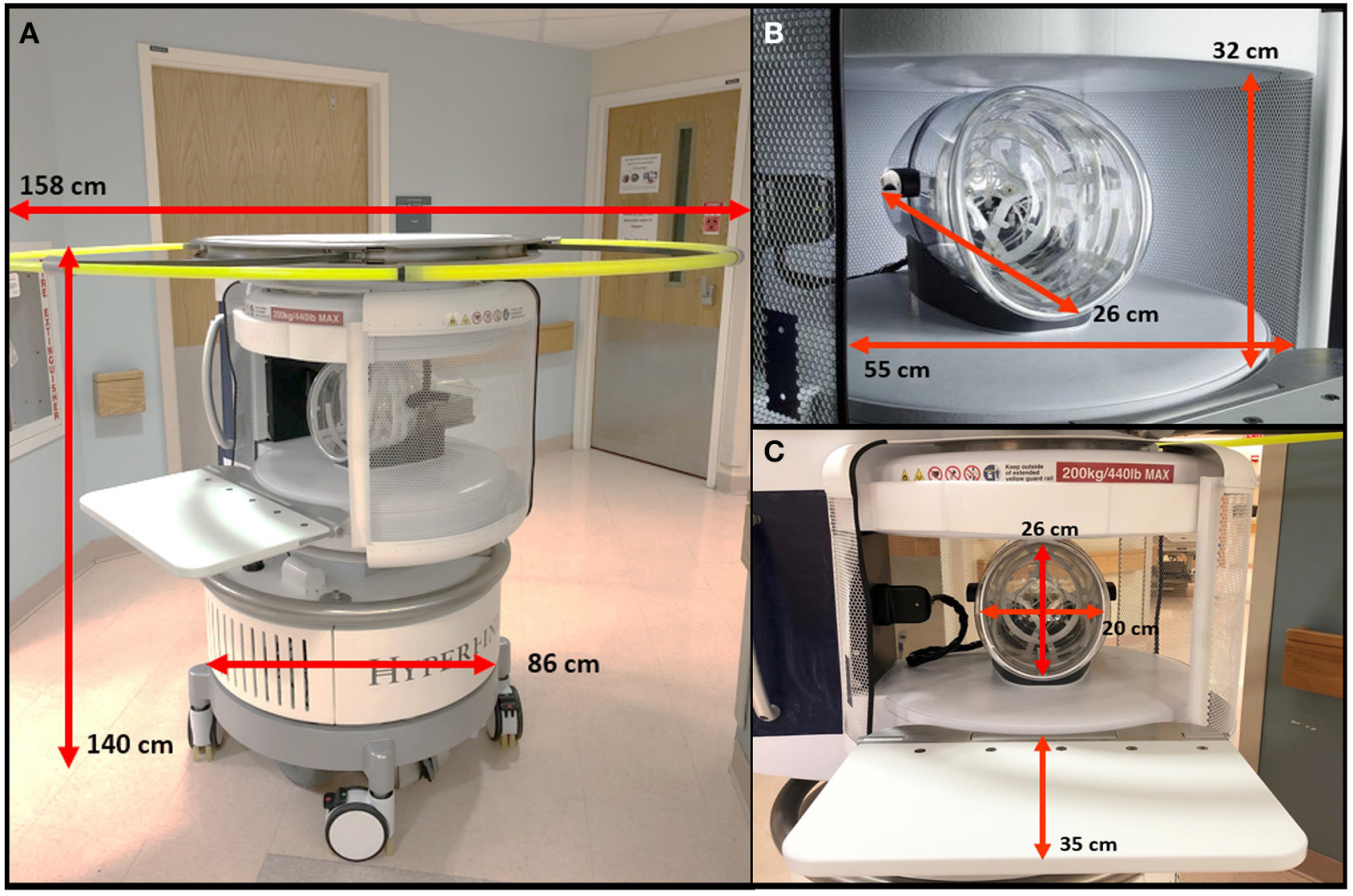
Portable MRI dimensions. **(A)** The pMRI device has a height of 140 cm and a width of 86 cm. There is an expandable ring Gauss Guard, with a diameter of 158 cm, which demarcates the 5 Gauss (0.5 mT) boundary. **(B)** The vertical space between the two magnets is 32 cm, within which there is a head coil of 26 cm length. The horizontal width between the RF shield, which is closed during scan acquisition, is 55 cm. **(C)** During a scan, the patient's head is positioned within the head coil (26 × 20 cm), and the scanner bridge (35 cm) is extended to adjoin with the patient's hospital bed.

All scans were conducted using a bedside pMRI system (Hyperfine, Inc., Guilford, CT, USA) which operates at 0.064T magnetic field strength. The device is cylindrical with a height of 140 cm, width of 86 cm, and weight of 630 kg. There is a 5 Gauss (0.5 mT) collapsible ring guard that extends from the top of the scanner into a circle with a diameter of 158 cm and is deployed when the device is in transit (Figure 1A). The scanner has a hinged bridge 35 cm in length which can be extended inside the patient's hospital room to adjoin with the head of the bed. The vertical space between the two magnets is 32 cm high, and the horizontal width between the closed RF shields is 55 cm. The head coil has the following dimensions: 26 cm long, 26 cm high, 20 cm wide (Figures 1B,C). The scanner is powered using a standard 15 A, 110 V wall outlet and does not require cryogens.

The scanning environment can contain standard ICU ferromagnetic equipment (including but not limited to the electrocardiogram and vital signs monitor, IV infusion pumps, IV stands, ventilators, compressed gas tanks, supply carts, and dialysis machines) but should remain outside the 5 Gauss line. The device allows for 3D, whole brain DWI, FLAIR, T2W, and T1W imaging. Table 1 lists acquisition times for all sequences available on the pMRI. Imaging protocols could be tailored to specific purposes or pathologies of interest. The device generates an acoustic volume level ranging from 60 to 80 dB within the head coil. The device uses a biplanar, unshielded gradient system which has peak gradient amplitudes of 25, 25, and 26 mT/m, on the X, Y, and Z axes, respectively, and gradient peak slew rates of 23, 23, and 67 T/m/s, respectively. Images were acquired by positioning the patient's head into a single channel transmit, 8-channel receive coil.

**Table 1:** Portable MRI brain sequences and acquisition times.

**Sequence**	**Acquisition time**
Pre-calibration sequence	01:03
Localizer	00:19
Auto-Align	00:31
Auto-Shim	00:07
FLAIR (AXI)	09:02
FLAIR (SAG)	08:27
FLAIR (COR)	08:10
T2W (AXI)	05:57
T2W (SAG)	04:55
T2W (COR)	05:27
DWI (AXI)	07:41
DWI with ADC map (AXI)	09:38
T1W (AXI)	05:38
T1W (SAG)	05:38
T1W (COR)	05:32

*FLAIR, fluid-attenuated inversion recovery; T2W, T2-Weighted; DWI, diffusion-weighted imaging; ADC, apparent diffusion coefficient; T1W, T1-weighted; AXI, axial; SAG, sagittal; COR, coronal*.

* *The listed times are for sequence acquisition. There may be additional time for processing/reconstruction*.

## Methods

This observational study was performed under an Institutional Review Board (IRB) protocol in accordance with guidelines and policies informed by the Yale Human Research Protection Program. Until August of 2020, pMRI exams were acquired after obtaining informed consent from the patient or their legally authorized representative. After this point, when the device was granted specific FDA approval, pMRI exams were obtained as part of the patient's clinical care without research consent.

### Screening

For patients admitted to the ICU or ED, we reviewed demographic data, clinical course characteristics, and available conventional neuroimaging from the electronic medical record. Depending on the type of pathology, clinical characteristics of interest included last known normal, time and date of surgical procedure, size of lesion, and location of lesion. For each patient, institution-specific MRI screening form, adhering to 1.5T MRI guidelines, was completed with the patient or the patient's legally authorized representative. Exclusion criteria included the presence of an MRI contraindication such as: pacemaker, defibrillator, implanted medication pump, vagus nerve stimulator, deep-brain stimulator, or programmable shunt; MRI-incompatible surgical hardware such as metal staples, screws, clips, etc.; suspected metal in eye; presence of spinal fractures. Patients who were pregnant during the time of hospital stay or <18 years old were also excluded. The pMRI device is safe for use with biomedical devices that have been cleared for magnets of field strength 1.5T and below.

### Coordinating

The bedside nurse was consulted to gauge each eligible patient's ability to lay flat for the duration of a pMRI scan. In the ED, the charge nurse was also consulted. Clinical reasons which limited a patient's tolerance in lying supine included intolerable back pain, elevated intracranial pressure, high risk aspiration, respiratory issues (e.g., desaturation, copious secretions, etc.), unstable blood pressure, and uncontrolled agitation. Some of these barriers were particularly relevant in patients with severe COVID-19 infection. If there were concerns for instability, the nurse performed a flat trial to see how the patient tolerated the supine position. A flat trial would allow the bedside nurse to assess if there may be a need for any additional sedation or analgesia needed for the scan and administer medications within ordered parameters.

A member of the clinical team (the attending physician, fellow, resident, physician assistant, or advanced practice registered nurse) was consulted prior to initiating the scan to ensure that there would be no interference with the patient's treatment pathway. This step was especially relevant in the ED, where patients admitted as stroke codes or intracerebral hemorrhage (ICH) alerts were often considered for emergent thrombectomy or surgical evacuation. If this was the case, pMRI scans were usually postponed so as not to delay those clinical interventions. In future deployments, pMRI could potentially be used as a valuable intraoperative tool during these time-sensitive procedures. If the attending physician desired an emergent pMRI exam, the scan was performed immediately. Otherwise, a time was scheduled with the patient's bedside nurse around other clinical procedures, including conventional neuroimaging, X-ray, echocardiogram, OR/IR procedures, physical therapy, and speech pathology.

### Transporting the Portable MRI

**Figure 2:**
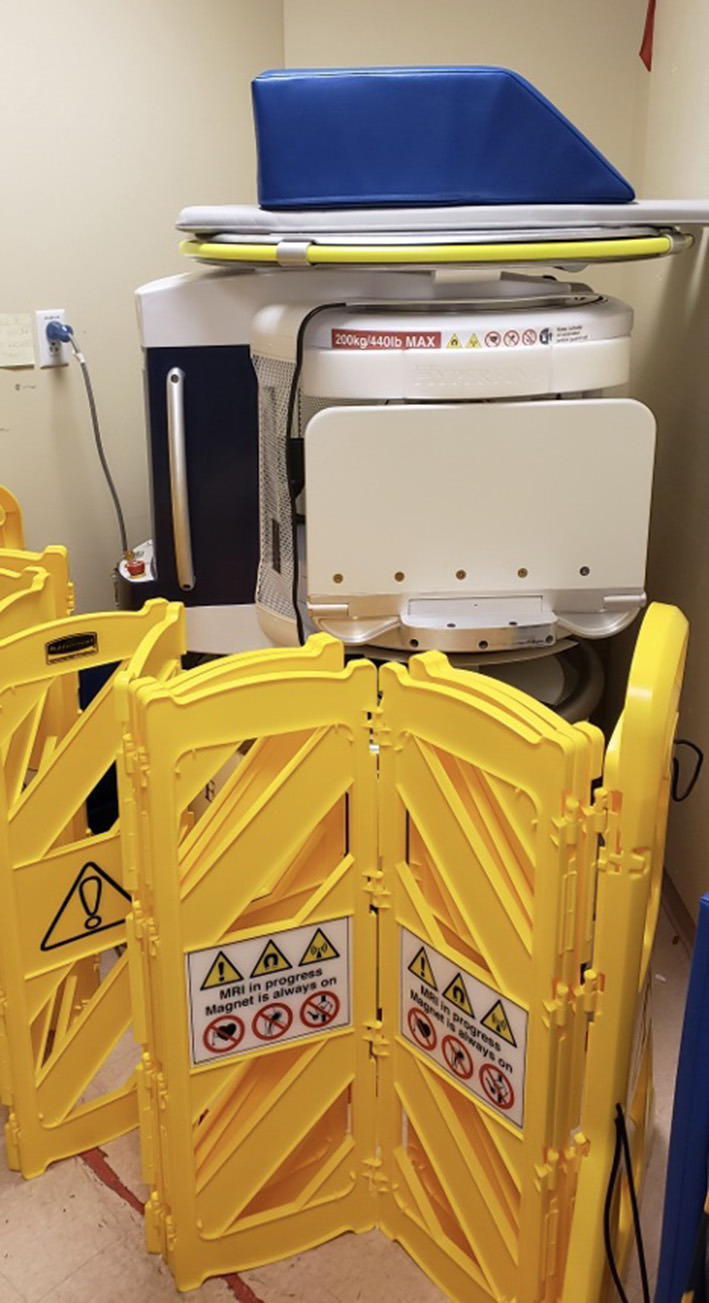
Designated storage of the portable MRI. The scanner is kept in a restricted-access equipment room requiring authorized badge access. The 5 Gauss line is delineated by cautionary barriers.

After a scan was coordinated, the pMRI device would be transported to the patient's hospital room from its secure storage location. In the YNHH ICU, it is kept in a restricted-access equipment room requiring authorized badge access with the 5 Gauss line delineated (Figure 2); in the ED at YNHH and the ICU at MGH, without any available restricted-access spaces, it is kept in a designated location away from the patient area provided by Hyperfine. Both locations contain cautionary signs that prohibit unauthorized access and indicate that the magnet is always on.

**Figure 3:**
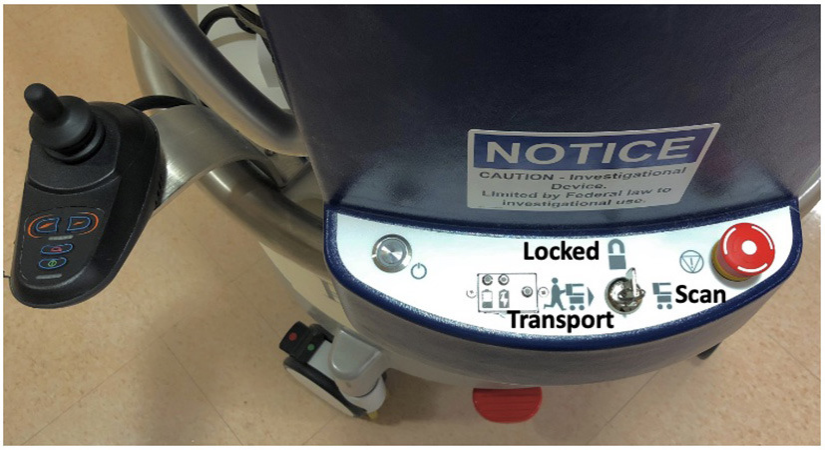
Portable MRI settings. The device has three settings, which can be selected using a key in the control panel. Transport mode (key positioned left) must be selected for the scanner to move, and a joystick on the left side of the control panel is used to steer. There are five speed settings, which can be adjusted by button. Locked mode (key positioned center) is used when the device is stationary and/or charging. Scan mode (key positioned right) is used during scan acquisition.

The pMRI device is powered by a motor and easily maneuvered by a single person. The device must be in “Transport” mode to move, with the key in the control panel turned to the left; there are also “Scan” and “Locked” modes, which are used for scanning and charging, respectively. There is a joystick located to the left of the control panel with buttons to adjust between five speed settings, depending on surrounding obstacles during transit (Figure 3). There are four wheels at the bottom of the device which provide 360-degree rotation for easy manipulation of direction. The high degree of mobility allows the device to be transported via elevator between different units and floors in the hospital. It maneuvers smoothly in both the forward and reverse directions. The Gauss guard, a collapsible ring that visually corresponds to the spatial extent of the 5 Gauss static magnetic field, was deployed whenever the device was in transit to protect any patients and personnel in the hallways and elevators. One operator steered the scanner while another operator walked in front to ensure a clear path. The scanner was able to fit through standard hospital hallways, transport elevators, and ICU door frames. The pMRI was not able to fit through door frames <86 cm in width. pMRI exams were conducted in the ICU and ED triage rooms due to accommodating size and single-patient occupancy.

### Executing a Scan

Prior to the examination, the scanner's head coil, magnets, bridge, and cushioning pads were cleaned and disinfected using Super Sani-Cloth Germicidal Disposable Wipes by PDI, Inc., in accordance with the manufacturer's instructions. To prepare the patient's hospital room, the bed was pulled 4 feet away from the wall to provide clearance for the scanner behind the head of the bed. The relative positions of IV stand(s), ventilator, physiologic bedside monitor, electrical and medical gas booms, and other equipment were adjusted in parallel with the bed so that any wires connected to the patient maintained adequate slack without being disconnected. All electronic equipment was kept outside of the controlled access area, as specified by the manufacturer. To ensure optimal access and safety, room furniture (i.e., bedside table, chairs, etc.) and personal effects/items were relocated to the far corners of the room. The television was turned off to reduce interference. Any IV bags hanging from the vertical metal poles at the head of the bed were moved to a mobile IV stand, and the poles were folded down. The scanner required a clear path unobstructed by wires, so the bed and IV cords were briefly unplugged if necessary to allow the scanner to clear. These were promptly reconnected after the scanner passed.

After the scanner was positioned at the head of the bed, it was plugged in and turned on. The scanner bridge was deployed, and the back cushion was put in place. This cushion provided padding for the back and shoulders when the patient was inside the scanner. The head of the bed was lowered so that the patient was supine. Prior to laying the patient flat, any nasogastric feeding tubes were placed on hold, and any external ventricular drains were clamped. The bed was adjusted so that it was level and flush with the bridge, and all transducers were re-leveled to allow for accurate monitoring during the exam. The bed was locked in place, and all restraints (arms, legs, abdomen) were undone, if applicable. If present, the patient's Foley catheter was moved from the end to the middle of the bed to avoid pulling or accidental disconnection. If the patient had Intermittent Pneumatic Compression devices attached to the legs, then they were disconnected for the duration of the exam. Any pillows were removed from underneath the patient's head, arms, and legs to aid in moving the patient into the coil. Any metal-containing accessories were removed including glasses, earrings, hair pins, necklaces, hearing aids, and dentures, as they often cause artifacts in the images acquired. Patients were offered ear plugs and an eye mask.

**Figure 4:**
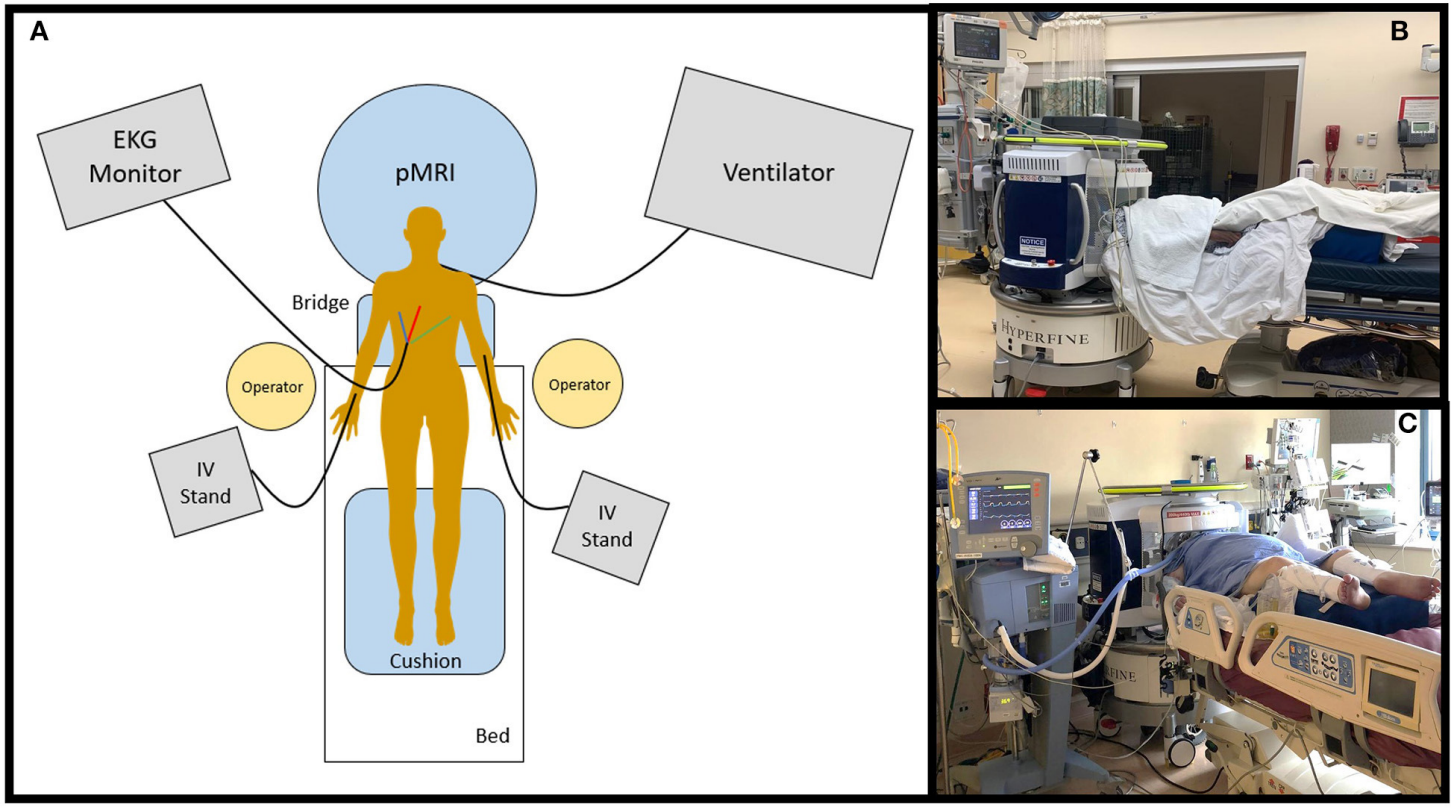
Portable MRI scan setup. **(A)** The pMRI was positioned behind the head of the patient's bed. The bridge was deployed so that the scanner adjoined with the bed, and the patient was positioned into the head coil. The pMRI was safe to use in the presence of ferromagnetic equipment, including an EKG monitor and leads, IV stand(s) and lines, ventilator and endotracheal tube, etc. These devices were not detached from the patient during scan setup or acquisition. At minimum, two operators were needed to position the patient into the scanner's coil. A cushion was provided underneath the legs to make the exam more comfortable. pMRI exams were conducted in the **(B)** emergency department and **(C)** intensive care units at Yale New Haven Hospital.

Once the bed and the scanner were both in the proper positions, the patient was positioned such that their head was as far back into the coil as possible. With at least one pMRI operator standing on either side of the bed, the bed-sheets underneath the patient were used to slowly move the patient backward until either the top of their head touched the back of the coil or their shoulders reached the base of the coil. The patient was moved in increments of ~10–15 centimeters using the draw sheet such that IV lines and stands could be moved in parallel, and any wires connected to the patient could be untangled. Optionally, a ceiling lift was used to position the patient in the coil then the draw sheet used ensure the patient was properly in the coil. Once the patient was positioned as deep into the coil as possible, a large cushion was placed underneath the legs to reduce any pain or pressure in the lower back (Figure 4). Pillows were replaced underneath the patient's arms to keep them supported and comfortable. Any restraints previously attached were re-tied. Foam cushions were placed on either side of the patient's face to limit movement of the head in the coil and to ensure it was positioned as straight as possible. The RF shield was pulled as fully closed as possible, either until it touched the bridge or the patient's left shoulder.

If alert and oriented, patients were informed that the best quality of images would be obtained if there was minimal movement inside the coil. They were also notified of the length of the scan and the kinds of noises to expect from the pMRI machine. In the absence of an emergency squeeze ball as with conventional MRI scans, operators remained inside or just outside of the room to make the patient aware of their presence and allow for the patient to communicate any discomfort or desire to be removed from the scanner. During the scan, operators regularly checked in with the patient verbally to avoid feelings of isolation and anxiety. Nurses and clinical staff were able to freely enter and exit the room during the exam to monitor vitals, administer and titrate medications, or draw blood. Cautionary signs were hung at the door to warn any pregnant staff or those with active, programmable implants of the magnet.

The scan was configured and run using an electronic tablet (iPad Pro, 2nd and 3rd generations; Apple) that could connect to the WiFi hotspot provided by the pMRI scanner. After logging into the Hyperfine interface, subject information was entered, and the desired sequences were selected. At minimum, the interface needed a Patient ID and year of birth to be entered before an exam can be initiated. The protocol ran until completion unless otherwise paused or terminated early. The exam could be paused at the end of each sequence, for re-positioning of the patient or other reasons, and restarted without issue. At any point during the exam, additional sequences could be added to the protocol, and sequence order could be altered.

**Figure 5:**
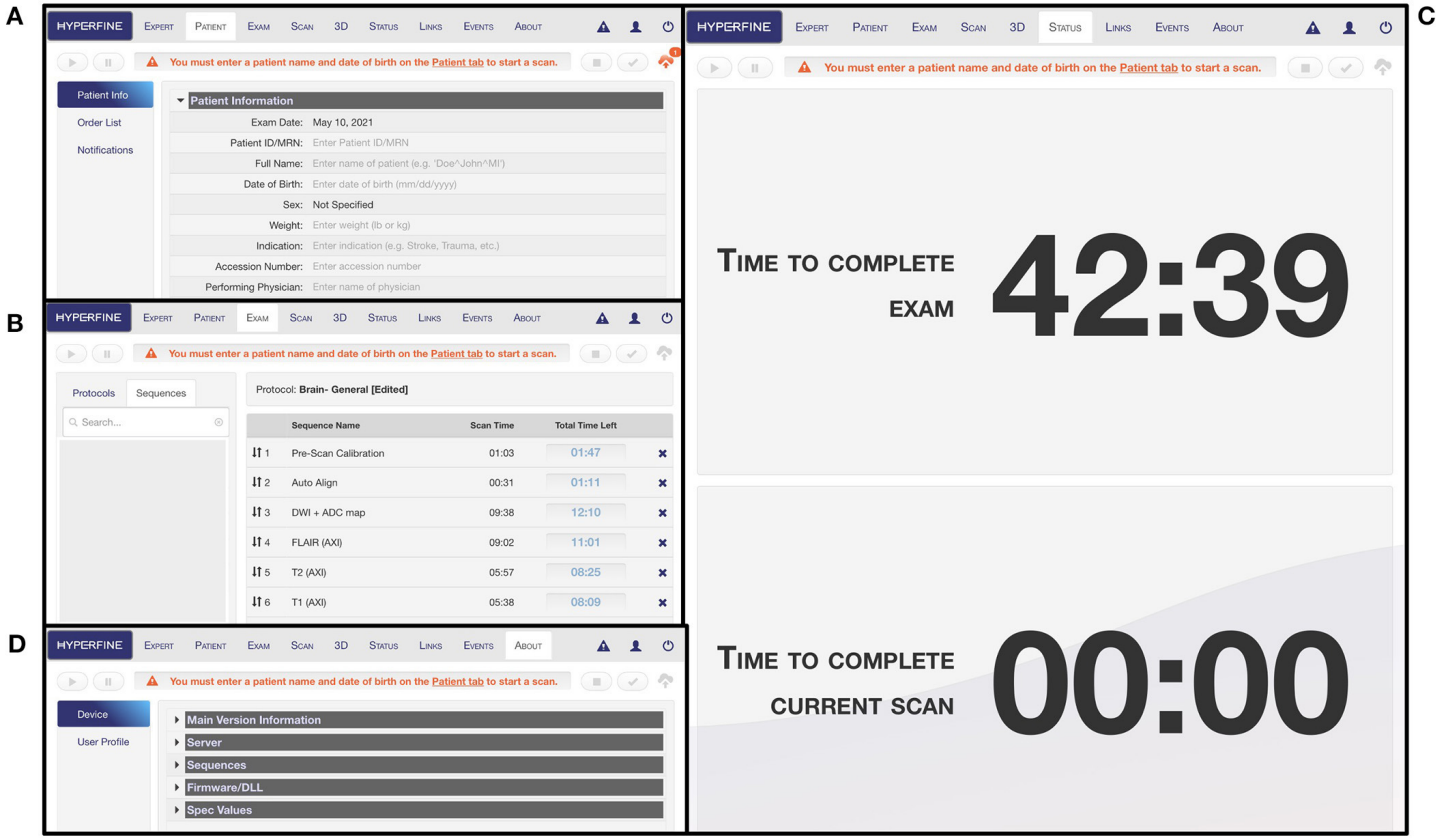
Portable MRI user interface. **(A)** The “Patient” tab is where patient information can be entered. At minimum, the patient ID/MRN and date of birth rows must be entered before an exam can be initiated. **(B)** The “Exam” tab is where the protocol can be designed with selected sequences. **(C)** The “Status” tab indicates the time remaining in the full exam as well as in the current scan sequence. **(D)** The “About” tab describes hardware and software information for the device.

**Figure 6:**
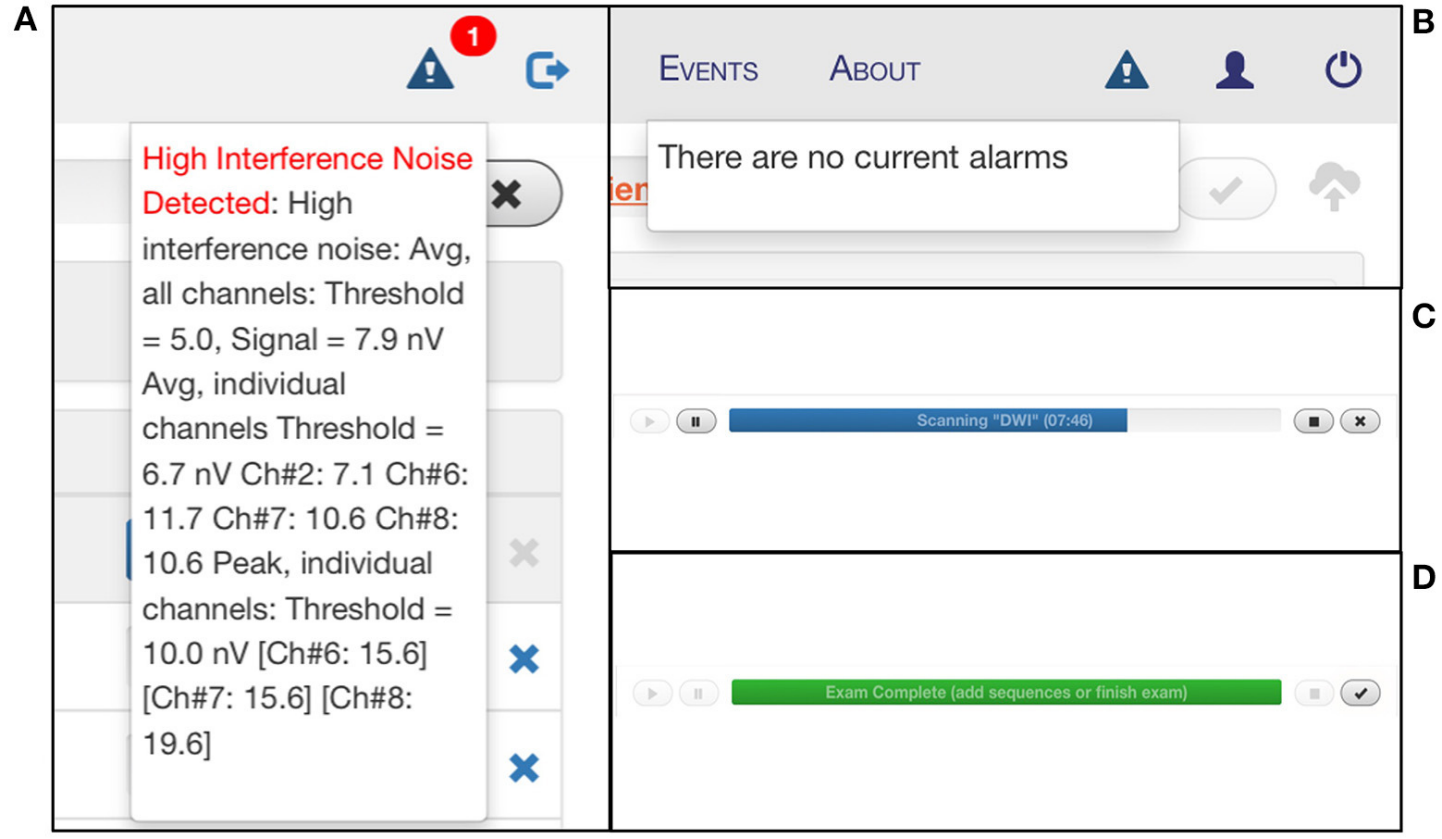
Monitoring portable MRI scan status. The triangular Alarm icon notifies the user of any error messages that arise during the scan, including “High Interference Noise” **(A)**, “Localizer Failure,” “Calibration Error,” or “No current alarms” **(B)**. **(C)** There is a status bar that indicates the time remaining in the current sequence and provides play, pause, stop, and cancel buttons. **(D)** At the end of the exam, the status bar turns green, and a check mark appears which can be pressed to upload the images to the Hyperfine Cloud as well as any other desired PACS system.

The interface has multiple tabs: the “Patient” tab is where patient information is entered (Figure 5A); the “Exam” tab is where desired sequences are selected (Figure 5B); the “Scan” tab is where the exam is viewed as images are acquiring in real-time; the “Status” tab contains a countdown of the time remaining in the full exam as well as the current sequence (Figure 5C); the “About” tab describes hardware and software details of the device (Figure 5D). There is a triangular icon at the top right corner of the interface that allows the operator to view alarm messages as the exam progresses, such as “High Interference Noise,” “Localizer Failure,” “Calibration Error,” or “No current alarms” (Figures 6A,B). There is a status bar at the top which notifies the operator how much time is left in the current sequence and contains Play, Pause, Stop, and Cancel buttons (Figure 6C). Upon completion of the exam, this status bar turns green, and a check mark appears which can be pressed to upload the images to the Hyperfine Cloud as well as any other desired Picture Archiving and Communication System (Figure 6D).

Our protocol consisted of a pre-scan calibration, localizer or auto-align, DWI with ADC map, FLAIR, T2W, and T1W sequences (42:39 min total including maximum processing time). If a patient needed to be removed from the scanner before completion of the full exam, the stop button was pressed on the interface. In case of an emergency, the red E-stop button on the scanner's control panel can also be pushed. Reasons for early termination included patient claustrophobia, agitation, discomfort, declining clinical status, transfer to another unit, or transport for another procedure.

When the patient was taken out of the scanner, cushions and pillows were first removed from around the patient's head, arms, and underneath the legs. Using the bedsheets or celling lift, the patient was slowly moved back into the bed, and any restraints were reapplied, if applicable. The bed was lowered to its original height, and the head was raised to a comfortable angle. The scanner was removed from the patient's room, and the bed was returned to its original position against the wall, along with any other objects and furniture that were moved. Outside, the scanner was sanitized again with disinfecting wipes and returned to its appropriate storage location. The device was plugged in to a standard outlet to charge and left on in “Locked” mode to allow for raw data to upload.

**Figure 7:**
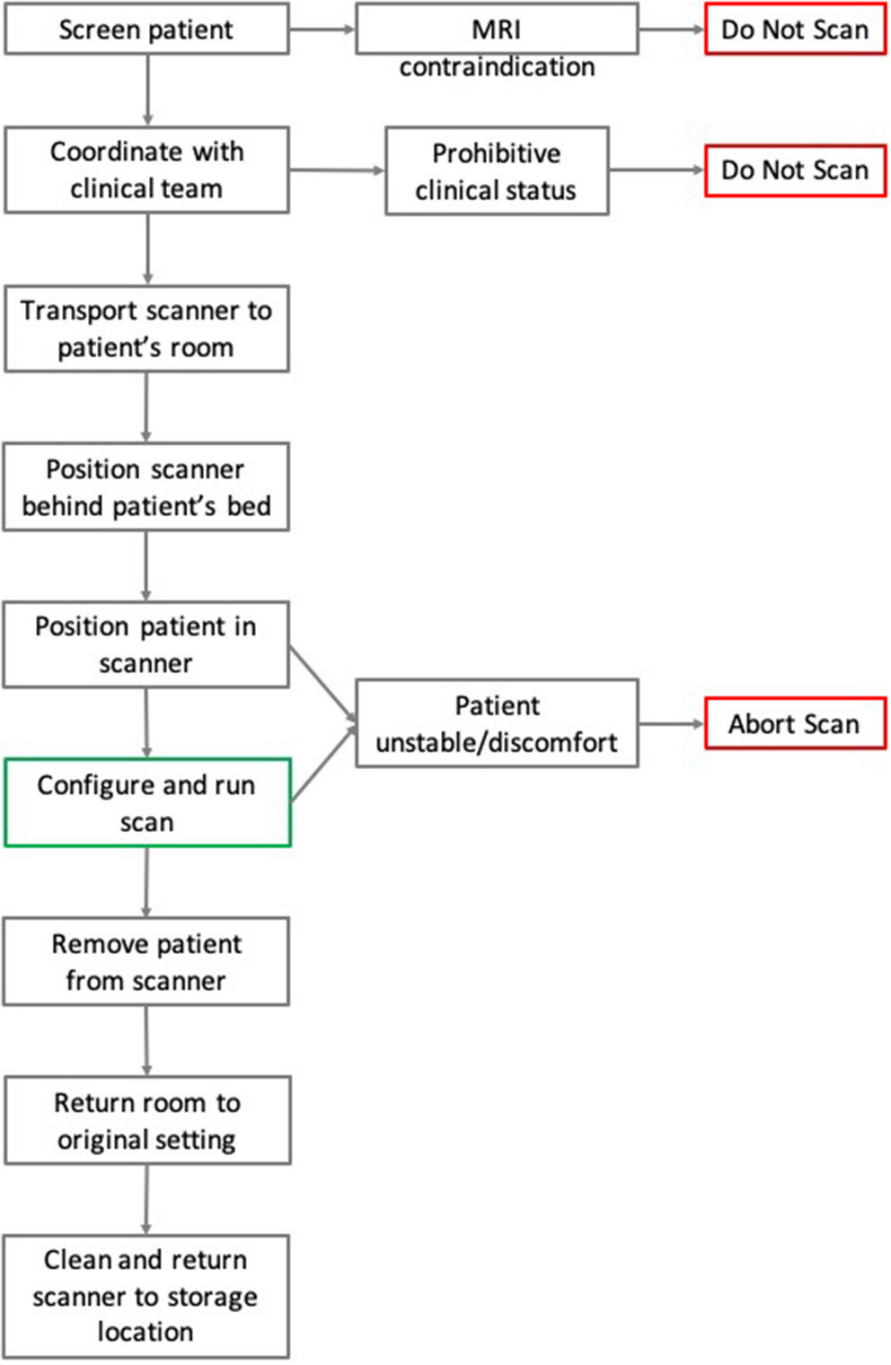
Flowchart of pMRI scanning methodology. Here the steps and chronology for the process of pMRI scanning are detailed. In the case of MRI contraindication or prohibitive clinical status, patients were not scanned. During a scan, if the patient suffered from instability or severe discomfort, then the exam was aborted.

The process for executing a pMRI scan is outlined in Figure 7.

## Results

### Specific Patient Populations: Considerations and Challenges

#### ^*^Obese/Elevated BMI Patients

Body habitus occasionally presented challenges for optimal positioning within the scanner's head coil. Factors that hindered positioning and depth included weight, head and nose size, shoulder width, neck length, and chest height. For patients suspected of possibly not fitting into the pMRI, the coil was removed and tested around the patient's head to determine if pMRI was plausible. Some helpful tactics employed for challenging patients included: (1) adjusting the bed into a Trendelenburg position allowing gravity to assist with the positioning if there was no concern for intracranial hypertension; (2) depending on the size of the patient, pulling their arms together around the torso to reduce shoulder width and allow them to pass through the RF shield. If the patient's head was large, or if they had many layers of head dressing after a surgery leaving minimal to no room in the coil, then the need for foam cushions on either side of the face was negated; (3) using the ceiling lift if available. Severely obese patients, especially those with tenuous respiratory status, would sometimes desaturate in the supine position, so their oxygen levels were continuously monitored throughout the scan. Our preliminary evidence has suggested that patients weighing up to ~140 kilograms can be successfully scanned; however, this rule is general and does not apply to all patients. The scanner has an official maximum weight limit of 200 kilograms, beyond which it is unsafe to scan.

#### ^*^Ventilated Patients

Ventilators were typically located on the side of the patient's bed closest to the hospital room door, while the bedside monitor and IV stand(s) were located on the opposite side away from the door. When the bed was moved 4 feet away from the wall to accommodate the scanner, the ventilator was moved in parallel to avoid accidental disconnection from the patient. Sometimes, it was useful to adjust the angle of the patient's bed in relation to the wall (rather than orthogonal) to maintain slack in the endotracheal tube and ventilator circuit. There were two methods for navigating the scanner to the head of the bed. One option was to disconnect the patient from the ventilator and temporarily connect to a portable oxygen tank with the help of a trained Respiratory Therapist (RT). This allowed the ventilator to be briefly unplugged from its power outlet and moved out of the way so that the scanner could pass behind. If the RT and/or the bedside nurse was not comfortable doing this, the other option was to bring the scanner around to the other side of the bed. In this case, the IV stand(s) were briefly unplugged to allow the scanner to pass, but the bedside monitor was not disconnected. Instead, the cord(s) were lifted vertically by one operator while the other maneuvered the scanner underneath. When the patient was being positioned inside the coil using the bedsheets, operators moved the patient in small increments so that the ventilator could be moved in parallel. Increasing the pMRI personnel to 3 or 4 people during positioning ensured that one operator had uninterrupted visualization of the endotracheal tube and ventilator tubing to minimize the risk of migration or dislodgement. For some patients, the endotracheal tube would occasionally point directly upward and hit the top magnet. If this was the case, the tubes were shifted to the corner of the patient's mouth to ensure that the head could remain straight (occasionally, this tubing precluded the ability to position the patient's head in the scanner altogether). After the patient was positioned, the ventilator was moved as far from the bed as possible—while still maintaining adequate slack in the endotracheal tube—and the monitor was turned away from the scanner's opening to reduce any interference noise. Throughout the setup and scan, the ventilator was kept outside of the controlled access area, based on the manufacturer's indications.

#### ^*^Patients on Dialysis

Patients who were connected to dialysis machines or continuous renal replacement therapy could be scanned without disrupting those processes. The same cautions were heeded as for ventilated patients regarding positioning the scanner in the room. If a patient had both a ventilator and dialysis machine located on opposite sides of the bed, then the scanner was positioned behind the bed via the side that required minimal disturbance of room equipment. If the room was larger, it was more convenient to bring the scanner around on the side of the dialysis machine. If the room was smaller, it was advantageous to bring the scanner on the side of the ventilator, since the dialysis machine should be kept outside of the 5 Gauss line of the pMRI device when running. Dialyzed patients often had a central venous catheter connected to the neck that was covered with thick dressing, which could not be disconnected or disrupted. If this was the case, the foam cushions placed on the side of the face were omitted to avoid disturbance.

#### ^*^Covid-19 Patients

Severe COVID-19 patients had multiple IV stands present in the room and were often ventilated and dialyzed due to multiple-organ dysfunction. When scanning patients with COVID-19 infection, a separate designated scanner was used to avoid cross-contamination in non-infected patients. Due to strengthened protocols by the hospitals, the entire surface area of the scanner was sanitized with bleach wipes before and after every scan. Operators of the pMRI wore the appropriate personal protective equipment required under hospital regulations, including surgical scrubs, N95 mask, face shield, disposable gown, bouffant cap, and nitrile gloves. These measures can also be generalized to patients with precautions for other infectious diseases, such as MRSA, E. Coli, VRE, etc.

#### ^*^Patients Post-thrombolysis or Mechanical Thrombectomy

After thrombolysis with tissue plasminogen activator or reperfusion by mechanical thrombectomy, patients required neurological assessments from the bedside nurse every 15 min for the first 2 h. These assessments included measurement of vital signs, pupil check, muscle strength, Glasgow Coma Scale score, and frequent National Institute of Health Stroke Scale scores. If these patients were scanned with pMRI, then one or more neurological checks needed to be omitted during the exam. This required written authorization from the patient's attending physician in the electronic medical record. Additionally, some thrombectomy patients were advised not to bend at the hip if it was a femoral access site for 2–6 h post-procedure (depending on use of closure device vs. manual pressure applied at the end of the procedure). The bedside nurse was consulted to assess whether it was safe to provide the patient with the cushion to elevate the legs after being positioned in the scanner.

#### ^*^Patients With Cervical Spine Precautions

Patients were sometimes put on cervical/spinal precautions if there was any suspicion of spinal injury, with examples including motor vehicle accident or possible trauma if found down. A cervical collar (C-collar) was often placed out of an abundance of caution, but the patient could still be scanned if there was no spinal fracture or immediate concern for injury. When scanning a patient with a C-collar, at least two people were needed on either side of the bed when sliding the patient into the scanner. This ensured that there was enough assistance to slide the patient very slowly and carefully without abrupt jerking movements. The process of removing the patient from the scanner followed the same method. One person gently held the patient's c-spine under the nape of the neck and guided the head into and out of the coil. While in the scanner, the patient was monitored closely for any signs of neck pain or tenderness.

#### ^*^Claustrophobic/Distressed Patients

Some patients who reported claustrophobia in conventional 1.5/3T MRI exams seemed to better tolerate the pMRI due to its open-geometry design and the fact that only the head is encased rather than the whole body. For others, claustrophobia or emotional distress during the exam were cited as reasons for early termination. For most patients, we did not administer sedatives or anxiety medications during pMRI scans, unless they were already intubated and receiving continuous sedation/narcotic infusion. Instead, we implemented other strategies to mollify patients and limit agitation, including offering eye masks and ear plugs, remaining in the hospital room during scan acquisition, and giving time updates between each sequence.

## Discussion

The pMRI is a useful tool not only because it can provide neuroimaging at the point-of-care, but also because it does so in a convenient and relatively low-stress manner. The device itself is easy to transport, position, and operate. It does not require patients to be disconnected from their equipment and limits the potential risks associated with traveling with critically ill patients and the conventional MRI magnet. It can function in the presence of common hospital ferromagnetic devices, including ventilators, IV pumps, EKG monitors, dialysis machines, and compressed gas tanks, outside of the 5 Gauss line. The pMRI is also a minimally intrusive device since the patient is not disrupted other than being positioned supine. The scanner plugs into a standard wall outlet and does not require a special power source or cryogens. It does not require a licensed MR technician to operate; instead, it can be operated by any non-credentialled staff member who undergoes simple training, thereby reducing the workload and inter-department cooperation that is typically required for transporting patients for neuroimaging. For these reasons, the ease and simplicity of pMRI render it useful in diverse clinical environments as well as outpatient clinics and, in the future, possibly ambulances.

A step in the right direction for point-of-care imaging can even be seen with pCT machines, which are currently deployed today in ICUs and may provide similar benefits as pMRI with regards to minimizing transport-associated risks. pCT offers neuroimaging at the patient's bedside, thereby increasing efficiency and providing safe radiological imaging while circumventing transport-associated hazards (13–15). For these reasons, pCT—particularly head and chest CT—can be extremely useful to critical care physicians, especially in the setting of cardiovascular, neurological, and/or respiratory instability and patients with extracorporeal lung assist (16). However, pCT involves several disadvantages when compared to pMRI. Usage of pCT in the ICU typically requires collaboration between trained CT technologists or radiology personnel, ICU nurses, and critical care technicians to operate the device and execute a scan (13, 15). Assembling a team with numerous people from different departments can be difficult and onerous to coordinate. Furthermore, CT imaging has notable shortcomings when compared with MRI, including its use of harmful radiation (17, 18). While efforts have been made to reduce radiation dose and burden in fixed CT scanners, this development has not been widely implemented in pCT. Another limitation is that CT does not allow for multimodal imaging with several different pulse sequences as MRI does. These sequences each emphasize different characteristics in the brain and can collectively provide a more thorough understanding of the pathology in question, with greater soft tissue contrast than is possible with CT (19–21). Finally, although MRI and CT have been found to be equal in their proficiency in detecting intracerebral hemorrhage, MRI has been shown to be superior to CT for detection of ischemia and restricted diffusion, especially in the hyperacute phase (19–22). The pMRI magnet, as an extension of MRI technology, therefore can be expected to provide a higher potential to assist diagnosis and/or treatment than pCT.

Perhaps the greatest utility of the pMRI lies in its ability to broaden the spectrum of patients who can receive MR imaging. By imaging at the bedside, patient populations who may otherwise be unable to transport can now be imaged. For example, we scanned 18 patients suffering from severe COVID-19 infection who did not receive any brain MRI or CT scans at all during their hospital stay. We also scanned an additional 17 COVID-19 patients who did receive conventional neuroimaging, but only after they had successfully undergone a pMRI bedside exam and had stabilized further for safe transport. In some cases, an incidental finding on pMRI aided patient care by detecting previously unknown pathology and giving clinical teams an indication of neurological status, which was especially advantageous for those patients who were sedated and paralyzed for ventilator management. Patients with other, non-COVID infectious diseases would also be prime candidates for pMRI because remote imaging at the bedside would enable contact precautions to be maintained, thereby limiting exposure to other patients and hospital staff. Besides COVID-19-related cases, there are other populations of critically ill patients who are too unstable to be transported from their hospital rooms and may benefit from pMRI imaging. Examples would include cardiac arrest patients, ventilated patients with severe acute respiratory distress syndrome, and those with multiple-organ failure who are connected to continuous renal replacement therapy.

**Figure 8:**
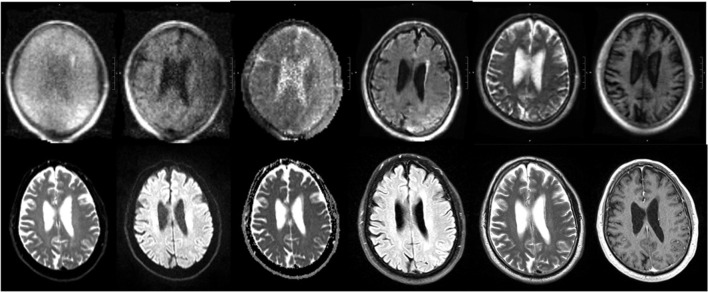
pMRI vs. conventional MRI. Sample pMRI images (top row) are provided along with the corresponding conventional imaging (bottom row). The sequences from left to right are: DWI (*b* = 0), DWI, DWI with ADC map, FLAIR, T2W, and T1W.

**Figure 9:**
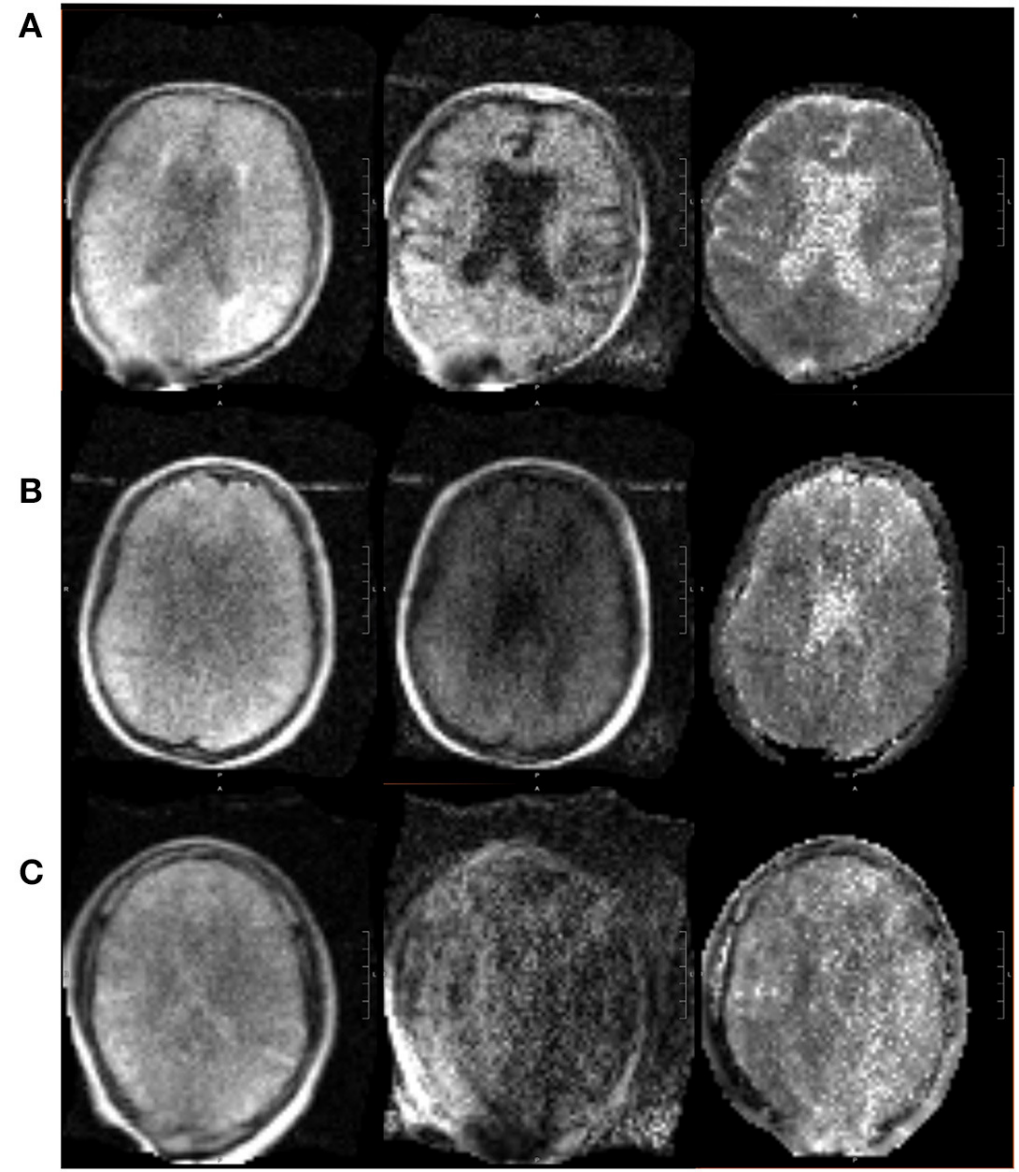
Range in pMRI DWI quality. Three sample DWI sequences are provided, of varying quality. From left to right: DWI (b = 0), DWI, DWI with ADC map. **(A)** In a relatively good scan, the sulci are appreciable, ventricles are distinct, and gray vs. white matter can be distinguished. **(B)** In an average scan, the sulci are less visible, and the ventricles are present but not clearly delineated. **(C)** In a poor scan, there is significant noise and pixelation such that structures cannot be adequately identified.

The pMRI has immense potential to change the current paradigm in neurological imaging. However, while the device can provide imaging at the point-of-care and increase the frequency of neurological monitoring, we acknowledge several limitations that currently obstruct its widespread clinical implementation. First, the total acquisition time is long compared to conventional MRI, requiring the patient to stay inside the scanner for upwards of 30 min when integrating FLAIR, T2, T1, and DWI with ADC map. However, the overall time frame for setup, scan acquisition, and removal is still less than that of a conventional MRI (when including time from order placement to completion and transport-associated delays). We have also seen significant reductions in scan times since the device was first deployed in 2018 as scanner upgrades improve the performance. Another drawback is the lower resolution of the images when compared with 1.5 or 3T MRI, a tradeoff that is inevitable with a low magnetic field strength. We have found that the image quality can be vulnerable to high interference noise from equipment in the hospital room as well as patient motion inside the scanner. Fortunately, software upgrades and processing reconstruction technology continue to be developed that can mitigate these factors and enhance the quality of the images. There have been significant improvements in quality since the device was first deployed at YNHH just 3 years ago, especially in the consistency and reliability of the FLAIR, T2W, and T1W sequences. A sample non-pathological exam is provided in Figure 8, alongside the corresponding conventional MRI. However, the DWI will require further improvements before the device can be used diagnostically as the image quality is more variable and susceptible to interference (Figure 9).

## Conclusion

We obtained neuroimaging at the bedside for patients in the ICU and ED at YNHH and the Neuroscience ICU at MGH. Our preliminary findings demonstrate the safety and feasibility of obtaining point-of-care MR neuroimaging in a wide range of patients who present with neuropathology. Our goal in this report was to describe the ease and convenience of operating the pMRI and incorporating it into the clinical workflow in various settings. We also sought to provide details on some of the barriers and challenges that we have encountered with respect to scan coordination and execution. While we acknowledge that there may be alternative ways of approaching pMRI scanning, our aim was to share the techniques that our team has found successful in different settings at YNHH and MGH. The methods presented here will likely adapt as this approach is used more frequently at multi-center institutions.

The pMRI is a novel solution that circumvents the need to disconnect patients from ferromagnetic monitoring devices or life-sustaining equipment for transport to conventional imaging suites, which thereby broadens the population of critically ill patients who can receive MRI. While pMRI is not used as a replacement for conventional CT and MRI at YNHH at this time, the device has expanded the potential for patients to receive serial imaging, which in turn helps clinical teams stay abreast of the status of their patients. Further work is still needed to deploy this technology in a larger variety of clinical settings to determine its full scope and utility.

## Data Availability Statement

The original contributions presented in the study are included in the article/supplementary material, further inquiries can be directed to the corresponding author.

## Ethics Statement

The studies involving human participants were reviewed and approved by Yale Human Research Protection Program. Written informed consent for participation was not required for this study in accordance with the national legislation and the institutional requirements.

## Author Contributions

KS takes responsibility for all independent decisions made regarding study concept and conclusions. AP and KS: design and writing of the manuscript. AC, MM, MY, IC, AW, WH, NT, SQ, JH, CW, GS, MSR, and WK: critical revision of the manuscript. AP, AC, MM, MY, and IC: data acquisition. All authors contributed to the article and approved the submitted version.

## Funding

This study was supported by funding from the American Heart Association (Collaborative Science Award 17CSA3355004, co-PI KS, WK, and MSR). The portable MRI device that was developed, deployed, and provided to Yale was borne out of an academic-industry collaboration, where the AHA grant supported the academic investigators. KS was supported by the NIH (U24NS107136, U24NS107215, R01NR018335, R01NS110721, R03NS112859, U01NS106513, and 1U01NS106513-01A1) and the American Heart Association (18TPA34170180, 17CSA33550004). The authors also declare that this study received funding from Hyperfine Research, Inc. The funder was not involved in the study design, collection, analysis, interpretation of data, the writing of this article or the decision to submit it for publication.

## Conflict of Interest

MSR is a founder and equity holder of Hyperfine, Inc. The remaining authors declare that the research was conducted in the absence of any commercial or financial relationships that could be construed as a potential conflict of interest.

## Publisher's Note

All claims expressed in this article are solely those of the authors and do not necessarily represent those of their affiliated organizations, or those of the publisher, the editors and the reviewers. Any product that may be evaluated in this article, or claim that may be made by its manufacturer, is not guaranteed or endorsed by the publisher.

## References

[1] Agrawal S Hulme SL Hayward R Brierley J. A portable CT scanner in the pediatric intensive care unit decreases transfer-associated adverse events and staff disruption. Eur J Trauma Emerg Surg. (2010) 36:346–52. 10.1007/s00068-009-9127-8 26816039

[2] Gimenez FMP de Camargo WHB Gomes ACB Nihei TS Andrade MWM Valverde M . Analysis of adverse events during intrahospital transportation of critically ill patients. Crit Care Res Pract. (2017) 2017:6847124. 10.1155/2017/6847124 29062574 PMC5618745

[3] Knight PH Maheshwari N Hussain J Scholl M Hughes M Papadimos TJ . Complications during intrahospital transport of critically ill patients: Focus on risk identification and prevention. Int J Crit Illn Inj Sci. (2015) 5:256–64. 10.4103/2229-5151.170840 26807395 PMC4705572

[4] Peace K Maloney-Wilensky E Frangos S Hujcs M Levine J Kofke WA . Portable head CT scan and its effect on intracranial pressure, cerebral perfusion pressure, and brain oxygen. J Neurosurg. (2011) 114:1479–84. 10.3171/2010.11.JNS091148 21166566

[5] Veiga VC Postalli NF Alvarisa TK Travassos PP Vale R Oliveira CZ . Adverse events during intrahospital transport of critically ill patients in a large hospital. Rev Bras Ter Intensiva. (2019) 31:15–20. 10.5935/0103-507X.20190003 30843950 PMC6443312

[6] Waydhas C. Intrahospital transport of critically ill patients. Crit Care. (1999) 3:R83–9. 10.1186/cc362 11094486 PMC137237

[7] Picetti E Antonini MV Lucchetti MC Pucciarelli S Valente A Rossi I . Intra-hospital transport of brain-injured patients: a prospective, observational study. Neurocrit Care. (2013) 18:298–304. 10.1007/s12028-012-9802-1 23208448

[8] Simmons A Hakansson K. Magnetic resonance safety. Methods Mol Biol. (2011) 711:17–28. 10.1007/978-1-61737-992-5_2 21279596

[9] Sheth KN Mazurek MH Yuen MM Cahn BA Shah JT Ward A . Assessment of brain injury using portable, low-field magnetic resonance imaging at the bedside of critically ill patients. JAMA Neurol. (2020) 8:e203263. 10.1001/jamaneurol.2020.3263 32897296 PMC7489395

[10] Mazurek MH Cahn BA Yuen MM Prabhat AM Chavva IR Shah JT . Portable, bedside, low-field magnetic resonance imaging for evaluation of intracerebral hemorrhage. Nat Commun. (2021) 12:5119. 10.1038/s41467-021-25441-6 34433813 PMC8387402

[11] Turpin J Unadkat P Thomas J Kleiner N Khazanehdari S Wanchoo S . Portable magnetic resonance imaging for ICU patients. Crit Care Explor. (2020) 2:e0306. 10.1097/CCE.0000000000000306 33381764 PMC7769347

[12] Zubair AS Crawford A Prabhat AM Sheth KN. Use of portable imaging modalities in patients with neurologic disorders: a case-based discussion. Cureus. (2021) 22:e15841. 10.7759/cureus.15841 34327077 PMC8301273

[13] Butler WE Piaggio CM Constantinou C Niklason L Gonzalez RG Cosgrove GR . A mobile computed tomographic scanner with intraoperative and intensive care unit applications. Neurosurgery. (1998) 42:1304–10; discussion 10–1. 10.1097/00006123-199806000-00064 9632189

[14] Gunnarsson T Theodorsson A Karlsson P Fridriksson S Bostrom S Persliden J . Mobile computerized tomography scanning in the neurosurgery intensive care unit: increase in patient safety and reduction of staff workload. J Neurosurg. (2000) 93:432–6. 10.3171/jns.2000.93.3.0432 10969941

[15] Peace K Wilensky EM Frangos S MacMurtrie E Shields E Hujcs M . The use of a portable head CT scanner in the intensive care unit. J Neurosci Nurs. (2010) 42:109–16. 10.1097/JNN.0b013e3181ce5c5b 20422797

[16] McCunn M Mirvis S Reynolds N Cottingham C. Physician utilization of a portable computed tomography scanner in the intensive care unit. Crit Care Med. (2000) 28:3808–13. 10.1097/00003246-200012000-00008 11153618

[17] Brenner DJ Hricak H. Radiation exposure from medical imaging: time to regulate? JAMA. (2010) 304:208–9. 10.1001/jama.2010.973 20628137

[18] Hricak H Brenner DJ Adelstein SJ Frush DP Hall EJ Howell RW . Managing radiation use in medical imaging: a multifaceted challenge. Radiology. (2011) 258:889–905. 10.1148/radiol.10101157 21163918

[19] Campbell BC Parsons MW. Imaging selection for acute stroke intervention. Int J Stroke. (2018) 13:554–67. 10.1177/1747493018765235 29543140

[20] Chalela JA Kidwell CS Nentwich LM Luby M Butman JA Demchuk AM . Magnetic resonance imaging and computed tomography in emergency assessment of patients with suspected acute stroke: a prospective comparison. Lancet. (2007) 369:293–8. 10.1016/S0140-6736(07)60151-2 17258669 PMC1859855

[21] Kidwell CS Chalela JA Saver JL Starkman S Hill MD Demchuk AM . Comparison of MRI and CT for detection of acute intracerebral hemorrhage. JAMA. (2004) 292:1823–30. 10.1001/jama.292.15.1823 15494579

[22] Shah S Luby M Poole K Morella T Keller E Benson RT . Screening with MRI for Accurate and Rapid Stroke Treatment: SMART. Neurology. (2015) 84:2438–44. 10.1212/WNL.0000000000001678 25972494 PMC4478034

